# COVID-19 Associated Illnesses From Alveoli to Glomeruli: A Case Report

**DOI:** 10.7759/cureus.25670

**Published:** 2022-06-05

**Authors:** Said Amin, Fawad Rahim, Mohammad Noor, Azhar Wahab, Sobia A Qureshi

**Affiliations:** 1 Internal Medicine, Khyber Girls Medical College, Peshawar, PAK; 2 Internal Medicine, Hayatabad Medical Complex Peshawar, Peshawar, PAK

**Keywords:** systemic hypertension, proteinuria, iga glomerulonephritis, acute kidney injury, iga nephropathty, corona virus disease 2019, sar-cov 2 infection

## Abstract

Hypoxemic respiratory failure is the most frequent complication of severe acute respiratory syndrome corona virus-2 (SARS-CoV-2) infection. Coronavirus disease-19 (COVID-19) is no longer considered a standalone respiratory infection. It can involve other organs, including kidneys by direct invasion or indirectly through immune activation, cytokine storm, microthrombi and hemodynamic instability. Multiorgan involvement carries a worse prognosis in COVID-19.

Tubulopathy is the most frequently reported renal pathology, followed by glomerulopathies. Among the glomerulopathies, immunoglobulin A (IgA) nephropathy is less often reported. Differentiating tubulopathy from glomerulopathy is important from the management and prognostic point of view. Laboratory investigations, including urine microscopy, cannot predict glomerulopathy as a cause of renal involvement. Therefore, it is important to proceed with renal biopsy early to make a definite diagnosis.

We report a case of a 33-year-old male who presented three weeks after recovery from COVID-19 with proteinuric acute kidney injury. Subsequent renal biopsy revealed IgA nephropathy.

## Introduction

Involvement of the respiratory system resulting in hypoxemic respiratory failure is the commonest manifestation of severe acute respiratory syndrome coronavirus-2 (SARS-CoV-2) infection [1]. Renal involvement is seen in up to 36.6% of cases of coronavirus disease-19 (COVID-19) [2]. The mortality of COVID-19 pneumonia is higher when there is renal involvement. It has been reported that acute kidney injury (AKI) develops more frequently in ventilated patients, and one-third of them die [3].

The risk factors for AKI are older age, diabetes mellitus, cardiovascular disease, preexisting chronic kidney disease, black ethnicity, hypertension, and the need for ventilation and vasopressor medications [4]. Multiple mechanisms have been postulated for renal involvement, such as direct cytotoxicity by the viral invasion of renal parenchyma through angiotensin-converting enzyme receptors, hemodynamic instability, microthrombi, drug toxicity, and cytokine-induced injury leading to renal tubular damage [5]. Reports from China and the United States of America (USA) on renal biopsies mostly show tubulopathies and rarely immune-mediated glomerulopathies such as collapsing focal segmental glomerulonephritis (FSGS), membranous nephropathy, and anti-glomerular basement membrane disease (anti- GBM disease) [6,7]. On review of 12 postmortem kidney biopsies of COVID-19 patients, only one was reported as IgA nephropathy [8]. Nicolas et al. reported a case of Henoch Schönlein Purpura (HSP) with IgA nephropathy in a young male with no prior history except COVID-19 [7]. Suso reported HSP & IgA nephropathy in a 78-year-old male with COVID-19 [9].

We report a case of a 33-year-old male who developed proteinuria and AKI following recovery from COVID-19 infection and who was found to have IgA nephropathy on renal biopsy.

## Case presentation

A 33-year-old man who was previously well presented with seven days history of fever, cough, and shortness of breath. He was diagnosed with a case of COVID-19 based on clinical presentation and a positive reverse transcriptase-polymerase chain reaction (RT-PCR) on a nasopharyngeal swab. He had mild SARS-CoV-2 infection as per the World Health Organization (WHO) classification for COVID-19 and received the appropriate treatment. His full blood count, C-reactive protein, urinalysis, liver, and renal function tests were unremarkable.

His symptoms improved significantly after two weeks. A week later, he developed weakness, headaches, and mild swelling of the feet. He visited the hospital again for further evaluation. His pulse was 84 beats per minute, blood pressure was 160/100 mmHg, temperature was 97F, and oxygen saturation was 96% on room air. He was having bilateral pitting pedal edema. There was no skin rash. The systemic examination was unremarkable. He was admitted for further evaluation. He had a full blood count, liver function tests, renal function tests, C-reactive protein, urinalysis, chest X-ray, electrocardiogram, and echocardiogram. Significant findings in the investigations are summarized in Table 1.

**Table 1:** Initial investigations at the time of admission g/dL: Gram/deciliter, mcL: Microliter, mg/dL: milligram/deciliter, IU/L: International unit/liter, ELISA: Enzyme-linked immunosorbent assay, HBsAg: Hepatitis B surface antigen, HCV: Hepatitis C virus, HIV: Human immunodeficiency virus, SARS-CoV-2: Severe acute respiratory syndrome coronavirus 2, PCR: Polymerase chain reaction

Investigations	Reference range	Results
Hemoglobin (g/dL)	13.5 to 17.5	12.4
Platelet count (x10^3^/mcL)	150 to 450	350
White cell count (x10^3^/mcL)	4.5 to 11	9.6
Neutrophils (%)	40% to 60%	54
Lymphocytes (%)	20% to 40%	36
Monocytes (%)	2% to 8%	09
Eosinophils (%)	1% to 4%	01
C-Reactive Protein (mg/dL)	< 0.5	0.131
Total Bilirubin (mg/dL)	0.2 to 1.2	0.2
Alanine aminotransferase (IU/L)	< 45	13
Alkaline phosphatase (IU/L)	< 350	70
Serum Albumin (g/dL)	3.4 to 5.5	3.1
Serum creatinine (mg/dL)	0.5 to 1.2	2.4
Urea (mg/dL)	20 to 40	43
PT (seconds)	12	12
APTT (seconds)	28	28
HBsAg (ELISA)	Negative	
Anti-HCV (ELISA)	Negative	
Anti-HIV (ELISA)	Negative	
SARS-CoV-2 PCR	Negative	
Urinalysis	+ protein, 3-4 pus cells	
Electrocardiogram	Within normal limit	
Echocardiogram	Within normal limit	
Chest X-ray	Normal	

Considering his symptoms, proteinuria and raised creatinine, an ultrasound abdomen and pelvis, a urine albumin creatinine ratio, anti-nuclear antibodies, anti-double-stranded deoxyribonucleic acid antibodies, serum C3 and C4 levels, anti-glomerular basement membrane antibodies, cytoplasmic-antineutrophilic cytoplasmic antibodies, and perinuclear-antineutrophilic cytoplasmic antibodies were advised. The results are summarized in Table 2.

**Table 2:** Subsequent investigations ACR: albumin creatinine ratio, ANA: antinuclear antibodies, Anti-dsDNA: anti-double stranded deoxyribonucleic acid antibodies, Anti-GBM: Anti-glomerular membrane antibodies, c-ANCA: Cytoplasmic-antineutrophilic cytoplasmic antibodies, p-ANCA: Perinuclear-antineutrophilic cytoplasmic antibodies

Investigations	Reference range	Result
24-hour urinary protein	Less than 150 mg/24h	884 mg/24 hour
Urine ACR	Less than 30mg/g	497 mg/g
ANA	Negative	Positive, titer: 1/80 Pattern: Cytoplasmic SPE
Serum C3 level (g/L)	0.8-1.6	1.31
Serum C4 level (g/L)	0.12-0.36	0.41
Anti-ds DNA	Negative	Negative
Anti-GBM	Negative	Negative
c-ANCA	Negative	Negative
p-ANCA	Negative	Negative
Ultrasound of abdomen and pelvis	Bilaterally normal size echogenic kidneys	

**Figure 1:**
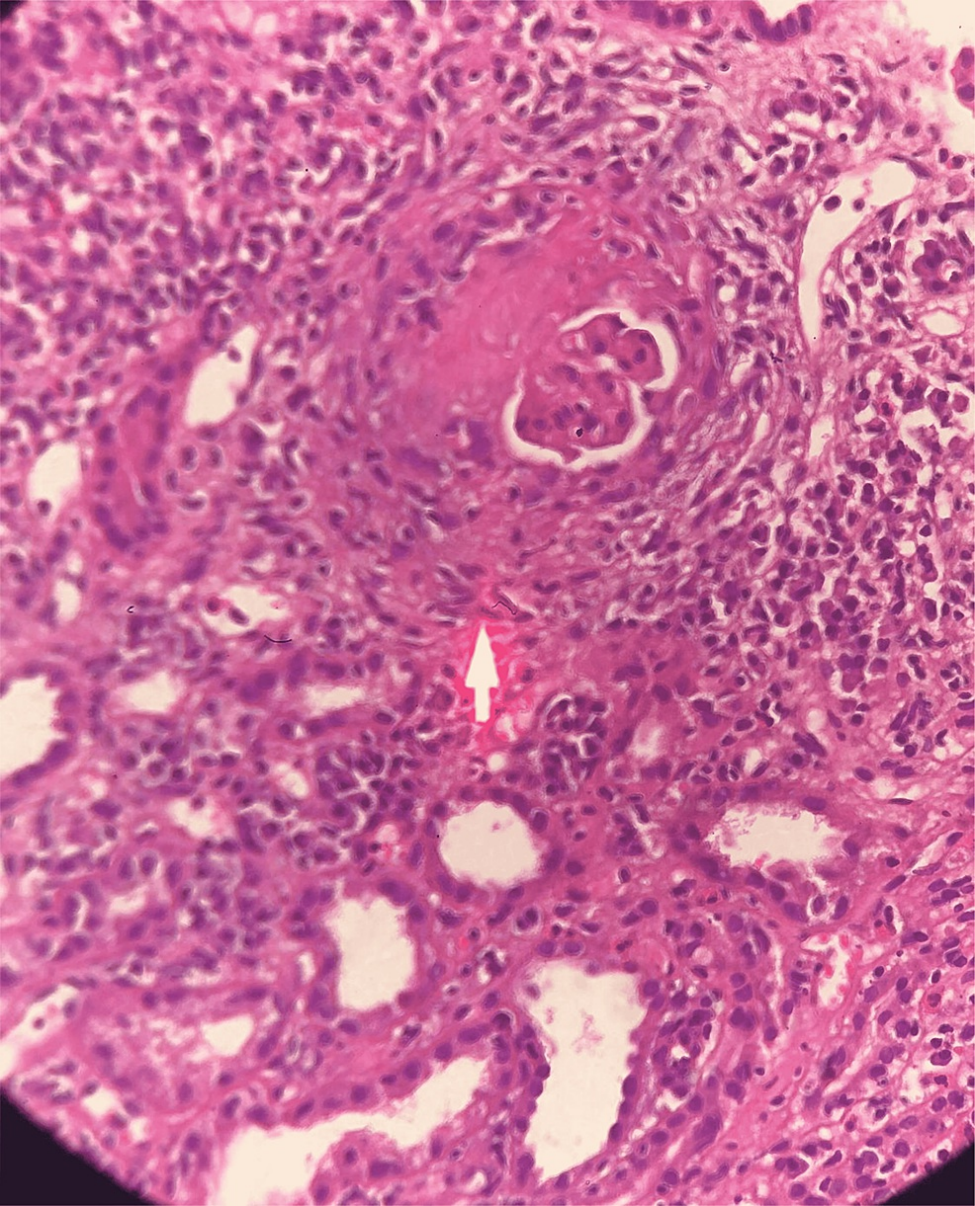
Renal biopsy (Eosin - Hematoxylin stain) showing focal segmental glomerulopathy.

**Figure 2:**
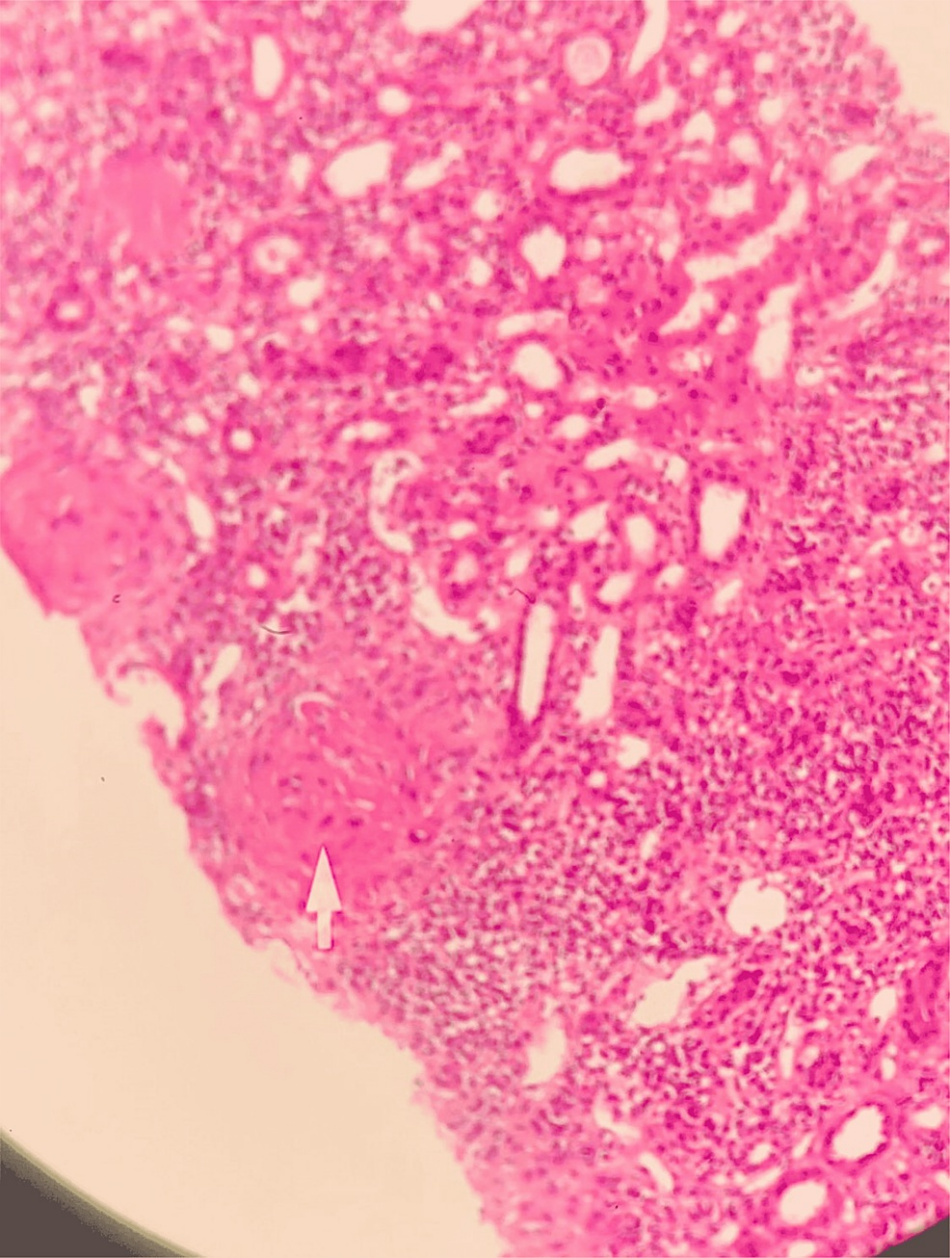
Renal biopsy (Eosin - Hematoxylin stain) showing glomerulosclerosis and edema of the tubular epithelia.

**Figure 3:**
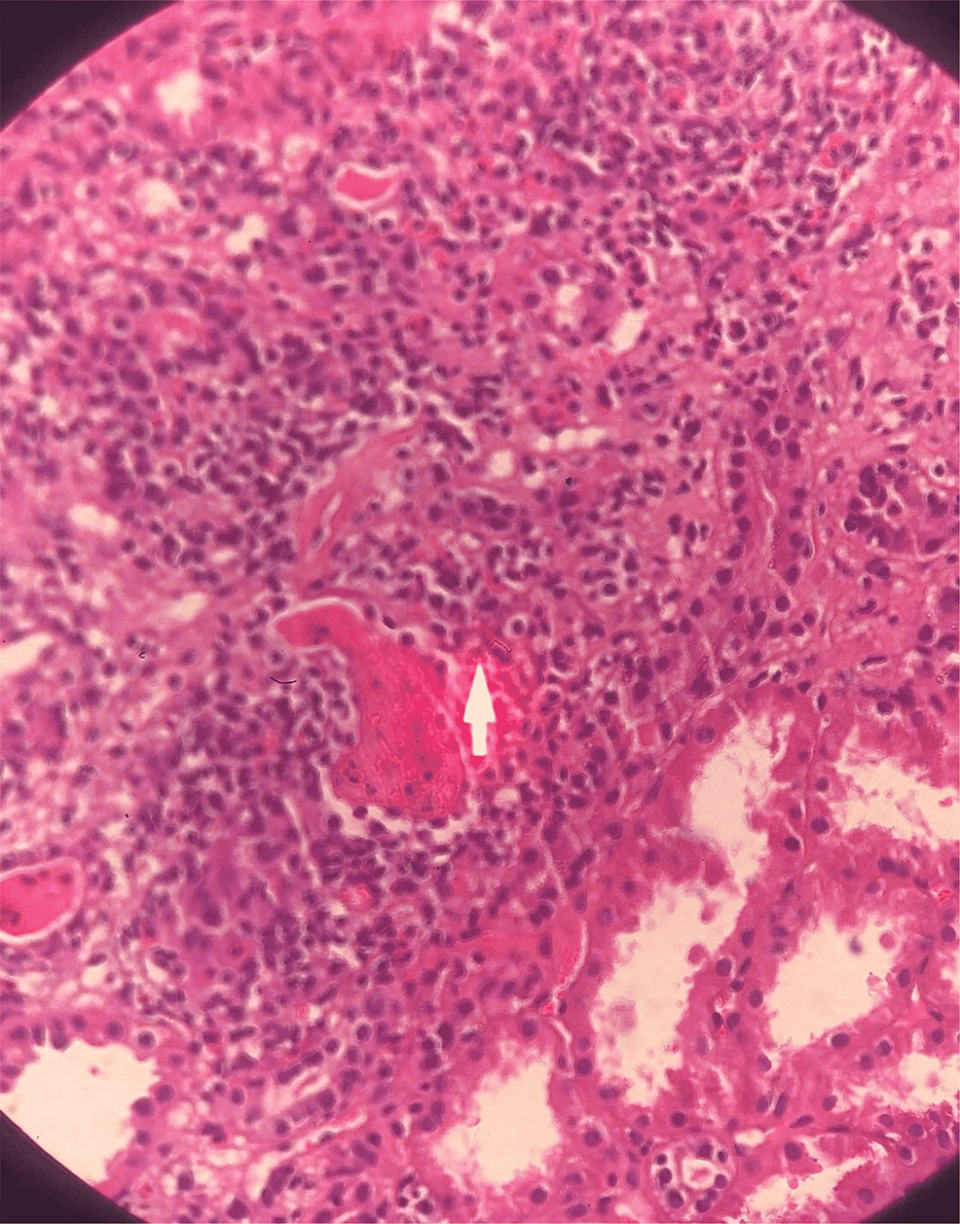
Renal biopsy (Eosin - Hematoxylin stain) showing tubulointerstitial inflammation.

**Figure 4:**
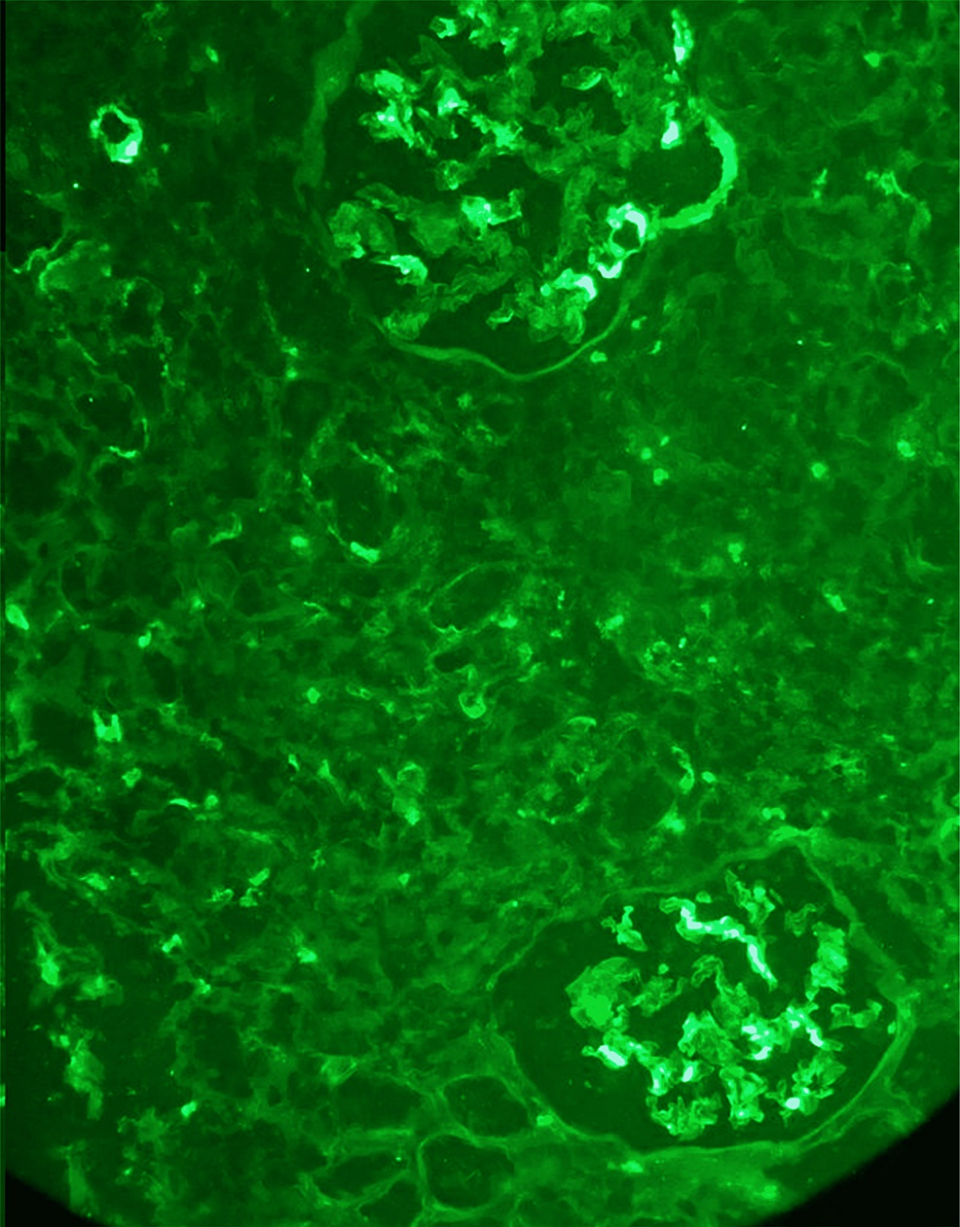
Renal biopsy (Immunofluorescence) showing IgA deposition in glomeruli and tubules.

Given the raised creatinine, proteinuria, and echogenic kidneys, he underwent an ultrasound-guided percutaneous renal biopsy. Pending the result of the renal biopsy, he was started on furosemide 40 mg daily and enalapril 10 mg daily. His edema disappeared, and his blood pressure remained 120/70 mmHg. The renal biopsy was reported five days after the procedure. It showed a total of eight glomeruli showing focal and segmental proliferation and inflammatory cells in the interstitium. The immunofluorescence revealed deposition of IgA in the glomeruli and renal tubules consistent with IgA nephropathy (Figures 1-4).

A multidisciplinary team, including a nephrologist, decided to continue the same treatment and to review him in four weeks. He was discharged from the hospital with the advice to restrict salt and regularly check blood pressure at home. He was reviewed in the outpatient department three weeks later. His blood pressure was 120/75 mmHg, pulse was 78 beats per minute, the temperature was 98F, and oxygen saturation was 98% on room air. There was no edema, and systemic examination was unremarkable. His serum creatinine was 1.2 mg/dl, urea was 36 mg/dl, and urinalysis showed trace protein. It was decided to withhold furosemide and continue enalapril. He is booked for the next outpatient visit in two months. He was advised to report early if he notices edema or worsening blood pressure.

## Discussion

The world’s healthcare paradigm has changed in a brief period of the pandemic of COVID-19. The notion that COVID-19 is a lung disease has changed soon with the discovery of the relationship between the ACE receptors and SARS-CoV-2 and the reports of other organ involvement worldwide.

The widespread presence of the ACE-2 receptors all over the body, which act as the primary entry point for the virus, provides the basis for the multi-system involvement in COVID-19 [10]. Kidneys are the new organs of interest for COVID-19 because of the widespread expression of ACE-2 receptors. Though hematuria, proteinuria, and deranged renal functions are the most common clinical presentations of renal involvement, the underlying pathophysiologic mechanism is multifactorial. Understanding the pathophysiologic mechanism of renal insult in COVID-19 is not straightforward. Though ACE receptors are expressed in renal tissue, there is controversy regarding direct toxicity by SARS-CoV-2. Other mechanisms postulated for AKI in COVID-19 include hemodynamic instability, drug toxicity, microthrombi in the renal vasculature, and cytokine-induced damage as part of the cytokine storm [11]. The SARS-CoV-2 induced cascade of events has been hypothesized to include circulating galactose deficient IgA1 (gd-IgA1) antibodies, formation of autoreactive antibodies to gd-IgA1, early conversion to IgA response, hyperactivation of IgA response in COVID-19 patients and development of immune complexes that deposit in various tissues such as skin, soft tissues, and kidneys [12].

Despite an initial hesitancy to perform autopsies due to fear of aerosolization of viral particles, many studies on postmortem biopsies published since May 2020 have highlighted different pathophysiologic, histologic, and immune-mediated patterns of COVID-19 related nephropathies [13].

Su H et al. from China reported the autopsy data of 26 cases. Almost all cases had acute tubular injury - loss of brush border, vacuolar degeneration and dilatation of tubular lumen with cellular debris, and nonspecific immunoglobulin M (IgM) and C3 trapping in glomeruli on immunofluorescence with corona-like particles on electron microscopy [14].

Tubulopathies were the most frequent histologic pattern in 46 postmortem biopsy reports stated by Dominick Santoriello et al. from New York, USA [8]. Sharma et al. from New York have also observed a few cases of immune-mediated glomerulopathies and thrombotic microangiopathies [15]. Larsen et al. from the USA presented a case report of Collapsing Glomerulopathy in a patient with COVID-19 [16].

We report an atypical and exceptional case of IgA nephropathy due to COVID-19. IgA-related vasculitis and IgA nephropathy are rarely reported to be associated with COVID-19 infection. Only 13 cases of IgA vasculitis with COVID-19 and four cases after COVID-19 vaccination have been documented so far [17]. Our case report is on a young male of 33 years who developed IgA nephropathy three weeks after COVID-19. These findings are consistent with the literature review, which shows a male predominance and the development of IgA nephropathy within 14 to 40 days of COVID-19 [18]. This case will add to the data about COVID-19-induced glomerulopathies, especially in developing countries like Pakistan. We acknowledge that electron microscopy was not performed on the specimens as this facility was not available locally.

## Conclusions

Although respiratory system involvement is the most frequent presentation of SARS-CoV-2 infection, concurrent renal involvement is not uncommon and is associated with poor prognosis. Renal involvement can happen at the time of COVID-19 symptoms and may develop several weeks after recovery from COVID-19. IgA nephropathy has been infrequently reported in association with COVID-19. The attending physician should be vigilant for renal involvement, and an early renal biopsy should be performed to make a definite diagnosis.
